# BCyrius: An Upgraded Version of Cyrius for Accurate CYP2D6 Genotyping From Short‐Read Sequencing Data

**DOI:** 10.1002/prp2.70065

**Published:** 2025-02-03

**Authors:** Andreas Halman, Rachel Conyers

**Affiliations:** ^1^ Cancer Therapies, Stem Cell Medicine Murdoch Children's Research Institute Parkville Victoria Australia; ^2^ Victorian Clinical Genetics Services Murdoch Children's Research Institute Melbourne Victoria Australia; ^3^ School of Population and Global Health, The University of Melbourne Melbourne Victoria Australia; ^4^ Department of Paediatrics The University of Melbourne Melbourne Victoria Australia; ^5^ The Novo Nordisk Foundation Centre for Stem Cell Medicine ReNEW, Melbourne Node Parkville Australia; ^6^ Children's Cancer Centre The Royal Children's Hospital Parkville Australia

**Keywords:** cyp2d6, genotyping, next generation sequencing, pharmacogenomics

## Abstract

Pharmacogenomics is a field of personalized medicine that aims to tailor drug dosing based on the genetics of an individual. The polymorphic and complex *CYP2D6* gene is important to analyze because of its role in the metabolism of approximately a quarter of all drugs. Several bioinformatic tools have been developed to genotype *CYP2D6* from short‐read sequencing data. Among these, Cyrius, a tool specifically designed for *CYP2D6* genotyping, has demonstrated high performance across various datasets. However, Cyrius has not been updated in the past 3 years, during which dozens of new star alleles have been identified and some previously defined ones revised. In this work, we simulated all known *CYP2D6* haplotypes to assess the ability of Cyrius to identify them. In that dataset, Cyrius was unable to call or misidentified 50 of 360 samples. Given the importance of providing an up‐to‐date tool, particularly in clinical settings, we present an upgraded version of the tool, named BCyrius, which includes all the missing star alleles as well as revisions to the previously listed ones. BCyrius successfully identified 100% of the currently defined minor star alleles, higher than Cyrius (85.6%) and the two other tested tools, Aldy and StellarPGx, which identified 92.2% and 87.8%, respectively. BCyrius also demonstrated slightly improved performance on a dataset of real biological samples, resulting in a higher call rate while maintaining similar accuracy with Cyrius. In addition to providing genotyping results, BCyrius also reports the predicted phenotype, along with information for each detected haplotype, including population frequencies.

## Graphical abstract

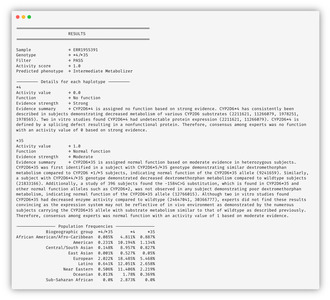

## 1 Introduction

Pharmacogenomics is a cornerstone of personalized medicine, utilizing genetic information to tailor drug dosing for patients [1]. Genetic polymorphisms in various drug‐metabolizing enzymes can significantly affect their function, potentially leading to changes in drug efficacy or adverse drug reactions [1]. Cytochrome P450 2D6 (CYP2D6) is a clinically important enzyme, metabolizing approximately 20%–25% of drugs, and has prescribing guidelines for at least 50 medications based on its activity [2]. However, pharmacogenetic analysis of the *CYP2D6* gene is challenging due to its high polymorphism and the presence of different structural variations (SVs) [3]. Several computational tools have been developed to genotype *CYP2D6* from short‐read sequencing data, and in our recent study, we benchmarked four of these tools, finding Cyrius to be the most robust with high accuracy in genotyping the *CYP2D6* gene [4].

However, Cyrius, an open‐source tool developed by Illumina, has not received public updates since mid‐2021, during which time dozens of new star alleles have been identified and some existing ones revised. Using outdated star allele definitions could lead the tool to report a wrong allele, potentially with a different function. For example, incorrectly identifying *18 as *1 would classify the allele as fully functional instead of the correct non‐functional status. This could affect the predicted phenotype, such as identifying it as a normal metabolizer instead of an intermediate or even poor metabolizer, which could have clinical implications. Therefore, it is important that a tool that can be used for clinical application is up to date to ensure the most accurate results and has the ability to identify rare alleles, which may be important in specific populations.

In this work, we first assess the performance of Cyrius using a simulated dataset that includes all currently defined haplotypes. After identifying the tool's limitations, we aim to upgrade it by revising the star allele definitions and addressing any issues in genotyping them. Finally, we validate these modifications using the same simulated dataset and also confirm the tool's performance on real biological whole genome sequencing (WGS) samples by comparing the results with the original version of Cyrius and with two other *CYP2D6* genotyping tools, Aldy [5] and StellarPGx [6]. These tools were selected for their demonstrated high performance in genotyping *CYP2D6*, alongside Cyrius [4], their ability to detect SVs [5, 6], and their recent updates within the past year.

## 2 Methods

The latest PharmVar database (version 6.1.3) was used to revise and define *CYP2D6* star alleles [7]. The ability to call star alleles was assessed by testing a tool on a simulated dataset of all known minor alleles (360 in total). Samples were simulated by first identifying the genomic regions used by the genotyping tools. To do that, we used the regions file utilized by Cyrius for determining star alleles and copy‐number variants (“CYP2D6_region_38.bed”) and extended all regions by 1 kb, followed by merging overlapping regions. Next, the *CYP2D6*‐*D7* gene region was manually extended to cover the entire complex without gaps. Control regions used by StellarPGx (*VDR* and *EGFR* genes) were manually added to the list, also with a 1‐kb extension. This final region file was then used to extract sequences from the GRCh38 reference genome, resulting in a new FASTA file to serve as the template for simulations.

To simulate all known minor alleles, the PharmVar (v6.1.3) dataset was downloaded from https://www.pharmvar.org/download, which includes the “CYP2D6.haplotypes.fasta” file containing *CYP2D6* sequences for all minor alleles. Each sequence was converted to its plus‐strand orientation and inserted into the template FASTA file, replacing the placeholder that was previously inserted for this purpose in place of the reference sequence. Next, the final template FASTA was used to simulate reads, followed by aligning FASTQ files to the GRCh38 reference genome using BWA‐MEM [8]. The aligned BAM files were then sorted and indexed with Samtools [9]. Samples were simulated to be homozygous for all variants to ensure that only the variants of the assessed star allele were present. All tools were run on the simulated samples using default parameters, except for Aldy, where we specified the control region as “chr12:47841537–47905022” (*VDR* gene). The *VDR* gene is considered an effective control gene due to its low copy number variation frequency and is used by StellarPGx [6]. The computational code used for simulation and running the tools, along with the regions file and FASTA template, is deposited on Zenodo [10].

With regard to the real biological samples, the tool was run on the 70 WGS samples of the Genetic Testing Reference Materials Coordination Program (GeT‐RM) dataset, as described in our earlier study [4]. To determine phenotypes, the activity scores and phenotypes for GeT‐RM samples were manually assigned using the allele functionality table from PharmGKB and Clinical Pharmacogenetics Implementation Consortium (CPIC) resources [11]. Based on the activity score (AS), the phenotypic groups were assigned as follows: poor metabolizers (AS = 0), intermediate metabolizers (AS = 0.25–1), normal metabolizers (AS = 1.25–2.25), and ultrarapid metabolizers (AS > 2.25).

## 3 Results

A total of 360 simulated samples, each representing a different *CYP2D6* minor star allele defined up to *175.001, were used to evaluate Cyrius's ability to genotype these alleles. Cyrius called 86.1% of the samples correctly, failed to make a call for 21 samples and incorrectly called 29 samples. Out of those, the function for 26 haplotypes differs from the simulated one (i.e., haplotypes with unknown/uncertain/unassigned function were reported as haplotypes with no/decreased/normal function, or when any of these latter three were assigned to an incorrect one, excluding unknown/uncertain/unassigned). Cyrius was unable to detect samples with star alleles *140 or higher, as these were not included in the tool's database. Additionally, Cyrius failed to correctly call some of the star alleles within its database, notably the *10.001 (*10A), as well as some non‐functional alleles such as *18, *20, *42, and *124, which were all detected as functional ones (either *1 or *2).

For comparison, two other pharmacogenomic genotyping tools were tested on the same dataset. Aldy (v4.6), currently the most up‐to‐date tool, includes star alleles up to *163, while StellarPGx (v1.2.7) covers alleles up to *145, both having a broader range of alleles than Cyrius. Aldy correctly genotyped 92.2% of the simulated samples, while StellarPGx correctly called 88.1%. The misidentification of *36.005 as *57 by Cyrius and StellarPGx was considered correct as *57 was its former designation with the same variant combination. The results for misidentified haplotypes by Cyrius and other tools are provided in Table 1, and individual calls along with indications of those resulting in a different function are listed in Table S1.

**TABLE 1:** Analysis results of BCyrius, Aldy, StellarPGx, and Cyrius on the simulated dataset.

Tool	Correct genotyping calls	Haplotypes with incorrect function	Haplotypes misidentified or not called
*BCyrius* (v1.0.2)	100%	0%	—
*Aldy* (v4.6)	92.2%	6.1%	*4.034, *15.003, *19.002, *36.001, *36.002, *36.003, *36.004, *36.005, *42.001, *83.001, *83.002, *83.003, *141.001, *150.001, *150.002, *151.001, *164–*175
*StellarPGx* (v1.2.7)	88.1%	10.3%	*6.004, *15.003, *20.001, *36.001, *36.002, *36.003, *36.004, *83.001^a^, *83.002^a^, *83.003, *141.001, *146–*175
*Cyrius* (v1.1.1)	86.1%	7.2%	*2.002, *4.034, *10.001, *15.003, *18.001, *19.002, *20.001, *36.004, *42.001, *124.001, *124.002, *128.001, *140–*175

*Note:* “Correct genotyping calls” show the proportion of correctly called homozygous haplotypes out of 360 total simulated samples, and “Haplotypes with incorrect function” represent the proportion of misidentified haplotypes that result in an incorrect function, potentially affecting the phenotype.

^a^ Denotes that the haplotype is present, but the diplotype is incorrect.

After identifying the incorrectly called star alleles in Cyrius, we developed an upgraded version of the tool, named BCyrius, which incorporates several modifications. First, we added all the missing star alleles up to the latest *175, based on the latest PharmVar database (v6.1.3) [7]. Next, we examined all instances where Cyrius either failed to make a call or made an incorrect call on samples containing star alleles previously listed in the tool, to identify the underlying reasons and implement necessary fixes. In most cases, we addressed the issues by modifying the star allele variant designations. However, some instances required further development, such as the ability to call *15.003 alleles, which none of the tools were able to do, likely due to the misalignment of reads to *CYP2D7* because of sequence similarity. Additionally, we slightly relaxed the genotyping model to allow for a small allelic imbalance when calling a variant in such cases. After implementing these modifications, we tested the final version of BCyrius on all the simulated samples and confirmed the ability to call all simulated haplotypes (100% concordance with the simulated diplotype and 100% call rate), outperforming the older version. BCyrius is also compatible with GRCh38‐aligned files that use both chromosome naming conventions (e.g., “chr1” and “1”).

In our previous work on benchmarking pharmacogenomics tools [4], Cyrius was tested on 70 real biological WGS samples, achieving 97.0% concordance with the ground truth and 95.7% call rate. BCyrius was then run on the same dataset (original samples with ~40× sequencing depth and those downsampled to 10×) to ensure it performed as well as the original version and the modifications did not introduce any performance issues. The upgraded tool produced identical genotyping results for all samples that Cyrius was able to call. Moreover, it successfully called two of the three original depth samples for which Cyrius did not provide a result, increasing the call rate to 98.6% while maintaining a nearly identical concordance of 97.1%. The diplotype concordance for BCyrius in samples without SVs was determined as 100% and for those with SVs as 90.9% (with 100% concordance for deletions and fusions, and 90% for duplications), which was also identical to Cyrius [4]. For comparison, StellarPGx showed a concordance of 94.3% and Aldy achieved 88.6% on the same dataset [4]. BCyrius correctly reported all phenotypes based on the identified haplotypes, with the exception of one case where “Indeterminate” was reported due to an incorrectly assigned haplotype, resulting in an overall phenotype concordance of 98.6%. However, since the only mismatched phenotype was “Indeterminate,” no phenotypes with incorrect metabolizer state were assigned. The phenotypic concordance for Aldy was 95.7% and 100% for StellarPGx. Aldy was the only tool to report phenotypes that differed from the ones assigned for the ground truth haplotypes, with two instances of discrepancy. The comparison results on the GeT‐RM dataset for all tools are provided in Table S2 (diplotypes) and Table S3 (phenotypes).

**FIGURE 1:**
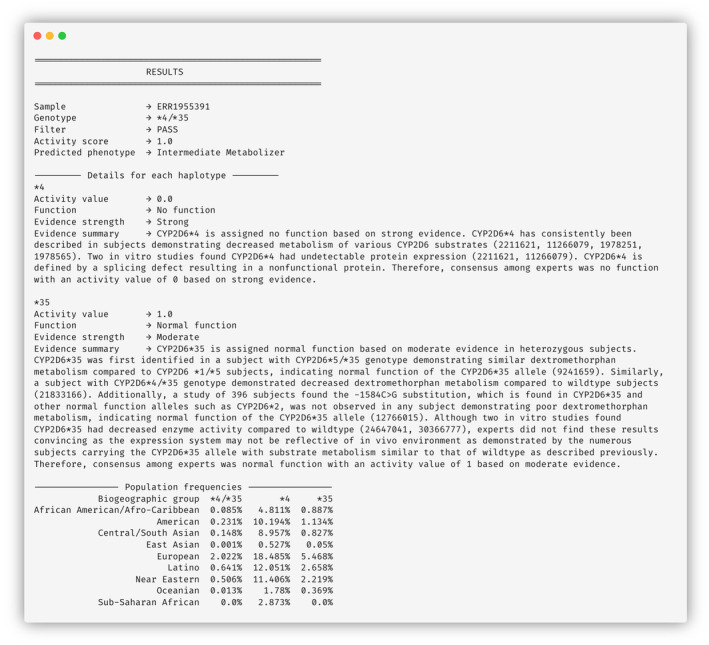
Example of output from an analyzed sample, including haplotype and population information.

Finally, while the original version of Cyrius provided only the genotype as output, we have integrated data from the CPIC/PharmGKB [11] into BCyrius and expanded the reporting to include the activity score, predicted CYP2D6 phenotype, haplotype information (activity value, function, strength and summary of evidence), as well as population frequencies for both the diplotype and each haplotype, thereby offering a more clinically relevant output. The example output containing all the information is shown in Figure 1.

## 4 Discussion

In this article, we introduced BCyrius, an upgraded version of the CYP2D6 genotyping tool Cyrius. The revised version resolved detection issues for 12 haplotypes and added the ability to correctly identify 38 haplotypes discovered in recent years, along with offering more clinically relevant output. BCyrius correctly identified all haplotypes in the simulated dataset and achieved 98.6% concordance for both diplotype and phenotype in the WGS dataset, surpassing the same metrics for the original Cyrius, Aldy, and mostly StellarPGx (with the exception of StellarPGx in phenotype concordance). More in‐depth assessment of the genotyping results from the latter three tools is discussed in our earlier article [4].

Using accurate and up‐to‐date tools is important, especially in clinical settings where genotyping results can influence therapeutic decisions. BCyrius not only maintains the high accuracy of the original Cyrius but also integrates the latest updates, enabling the detection of more rare star alleles. Additionally, BCyrius provides accurate phenotype predictions, along with information on detected haplotypes and population frequencies, which can aid in interpreting the results. Ultimately, with the provided updates, BCyrius has the potential to improve patient care. We aim to maintain the tool and provide updates as new star alleles are discovered or revised, with potential improvements for detecting rare complex alleles, ensuring BCyrius remains a reliable tool for *CYP2D6* genotyping. The tool is open source and freely available at https://gitlab.com/andreassh/bcyrius.

## 5 Limitations

All samples were aligned using the BWA‐MEM algorithm without any post‐alignment processing. Using a different aligner or incorporating post‐alignment processing steps could affect downstream analysis and potentially lead to different results. In this work, simulated data were used to assess the ability of tools to correctly identify star alleles. While this can also indicate the accuracy of genotyping homozygous star alleles, it does not fully reflect performance for heterozygous alleles, which are significantly more complex to genotype due to phasing and depend on the method used by the tool. A dataset of real biological samples was used to determine concordance with the ground truth; however, a larger dataset with more variable diplotypes, especially those comprising haplotypes that share variants, would provide a more accurate estimate. In BCyrius, support for the older GRCh37 reference genome has been discontinued, and the tool is now compatible only with the GRCh38 assembly.

## Author Contributions

A.H. and R.C. wrote the manuscript; A.H. designed and performed the research, software development, and analyzed the data, with input from R.C.

## Conflicts of Interest

The authors declare no conflicts of interest.

## Supporting information


**Table S1.** Genotype calls for simulated samples.
**Table S2.** Genotype calls for real biological WGS samples.
**Table S3.** Predicted phenotypes for real biological WGS samples.

## Data Availability

All data generated during this study are included in this published article and its supplementary information files. The data and computational code used for simulations and genotyping are available on Zenodo at https://doi.org/10.5281/zenodo.13357382. All analyzed GeT‐RM samples can be accessed through the European Nucleotide Archive (project: PRJEB19931). The source code of BCyrius is available at https://gitlab.com/andreassh/bcyrius.
